# European Health Technology Assessment Considerations Related to Gene Therapies in Eyecare: The Neovascular Age-Related Macular Degeneration Example

**DOI:** 10.3390/jmahp13030042

**Published:** 2025-08-27

**Authors:** Kevin Douglas, Gianni Pardhanani, Laetitia Mariani, Maria Chaita

**Affiliations:** 1 AbbVie, Chicago, IL 60064, USA; 2 AbbVie, 00144 Rome, Italy; 3 AbbVie, 6330 Cham, Switzerland; 4 AbbVie, Marlow SL6 4UB, UK

**Keywords:** anti-vascular endothelial growth factor (anti-VEGF), European Health Technology assessment (EU HTA), real-world evidence (RWE), indirect treatment comparisons (ITC), gene therapies, eyecare, European Access Academy (EAA), neovascular age-related macular degeneration (nAMD)

## Abstract

Gene therapies that induce the body to produce therapeutic anti-vascular endothelial growth factor (anti-VEGF) proteins are an emerging topic related to neovascular age-related macular degeneration (nAMD). Continuous delivery of anti-VEGF protein directly to the target tissue offers the possibility of lifelong efficacy without the need for repeated and frequent eye injections. This novel approach could revolutionize patient management through optimizing clinical outcomes while simplifying service delivery. However, such gene therapies are anticipated to face unique challenges related to patients’ access and health technology assessment (HTA), and their integration into real-world eyecare practices. This article presents key elements raised at the European Access Academy (EAA) Fall convention (held in Rome in October 2024) regarding anticipated HTA challenges for gene therapies in nAMD. The important role of HTA and policymakers in ensuring that emerging gene therapies are accessible to all eligible patients is also highlighted. This article mainly focuses on the need for a fit-for-purpose EU HTA framework to address the widely varying utilization of standard of care in nAMD clinical practice, and to incorporate considerations about the long-term durability of gene therapies in nAMD. The importance of integrating real-world evidence (RWE) into the EU HTA framework is also discussed.

## 1. Introduction

Age-related macular degeneration (AMD) is a chronic, progressive ocular disease characterized by damage to the macula, the central region of the retina [1]. Neovascular AMD (nAMD) is a subtype of AMD and is responsible for approximately 80% of the severe vision loss caused by AMD [2]. nAMD has a severe impact on patients’ eyesight, causing visual distortions, central vision loss, blurred vision, and difficulty perceiving color, and may ultimately lead to blindness. nAMD has a profound impact on patients’ lives, as individuals with this condition lose their ability to see fine details, read, drive, and recognize faces [2].

nAMD is currently managed with frequent anti-vascular endothelial growth factor (anti-VEGF) injections into the vitreous of the eye [3]. These anti-VEGF therapies were originally investigated when administered via fixed continuous regimens (e.g., monthly or bimonthly intravitreal injections), and their efficacy has been demonstrated in various randomized clinical trials (RCTs) [4,5,6]. In practice, treatment is generally administered on an individual patient basis according to either a pro re nata (PRN) or treat-and-extend (T and E) regimen, to reduce the number of injections and monitoring visits required [7,8]. T and E is currently considered the gold standard, as meta-analyses of RCTs comparing T and E vs. fixed regimen protocols have indicated T and E to be as effective as fixed-dose regimens when implemented strictly within the context of an RCT [9]. However, aVEGFs often lead to suboptimal results in real-world settings vs. the evidence from RCTs, mainly driven by lack of adherence or differences in clinical settings across countries [10].

Gene therapies are being investigated for the treatment of nAMD with the potential to address these shortcomings through the continuous delivery of an anti-VEGF protein that could minimize treatment burden and enhance long-term patient outcomes. Gene therapies could represent a promising advancement in the treatment options for nAMD [11]. However, unique challenges to access and health technology assessment (HTA) are anticipated for gene therapies highlighting the need for fit-for-purpose HTA frameworks. 

Regulation (EC) No 1394/2007 on advanced therapies (ATMPs) is designed to facilitate their access to the European (EU) markets, while guaranteeing the highest level of health protection for patients [12]. Advanced therapies are new medical products that use gene therapy, cell therapy, and tissue engineering [13]. Gene therapies contain genes that lead to a therapeutic, prophylactic, or diagnostic effect, and usually treat a variety of diseases (including genetic disorders, cancer, or long-term diseases) [14]. Following the entry into force of the EU Health Technology Assessment Regulation (EU HTAR) [15], ATMPs undergo Joint Clinical Assessment (JCA) since January 2025. The EU HTAR aims to strengthen the quality of HTA across the EU and to contribute to improving the availability of innovative therapies to EU patients [16].

Although the intention of the EU Regulations is to facilitate access of innovative therapies to European patients, access to gene therapies could face challenges due to uncertainties raised during the health technology assessments (HTAs), primarily due to their innovative nature and the emerging evidence base. Evidence of long-term outcomes and durability of effect from a one-time injection, as well as robust evidence from real-world settings—much of which will not be available for these ATMPs at launch—represent some of the issues which fit-for-purpose HTA frameworks should address.

The HTA-related challenges that gene therapies may face were described during the European Access Academy (EAA) Convention breakout session on ‘Case Study—Gene Therapies & ATMPs—Ophthalmology’ (hereafter referred to as the EAA breakout session) that was held in Rome in October 2024 (EAA Convention information available at: https://www.euaac.org/ (accessed on 24 January 2025)). Multiple stakeholders attended the EAA breakout session, namely, patient association representatives, health economics professors, HTA experts, and pharmaceutical industry representatives (including gene therapy developers like AbbVie) (~12 attendees in total). During the EAA breakout session, the example of gene therapies in eyecare, specifically in nAMD, and the associated EU HTA challenges for the assessment of gene therapies were discussed. This article provides a summary reflecting the overall views on key discussion points raised during the EAA breakout session.

## 2. Key Considerations from EAA Breakout Session for Joint Clinical Assessment of Gene Therapies in nAMD

A. A clear framework for assessment of long-term durability and the use of Real-World Evidence (RWE) is critical for gene therapies in nAMD.

○ Gene therapies may lack extensive long-term outcomes at the time of launch.
  ▪ JCA should leverage evidence-based scientific information that supports a reasonable assessment of durability beyond the time period of the randomized clinical trial (RCT), which is particularly relevant for one-time gene therapies with the potential for life-long efficacy.
  ▪ Open-label uncontrolled long-term follow-up (LTFU) data should be accepted as a useful source of supportive evidence.
  ▪ Post-HTA RWE should be leveraged to validate assumptions around the durability of the effect of gene therapies (i.e., through an update of the initial JCA).
  ▪ EU HTA should capture the perspective of all relevant stakeholders and ensure that expert opinions are used to support durability arguments for gene therapies.

Unlike traditional therapeutics, gene therapies promise long-term benefits often with a single dose. However, at the time of launch, there may be limited evidence on the long-term benefits of such therapies. The uncertainty around the long-term outcomes of gene therapies creates challenges in the assessment of their added clinical value by HTA [17]. In addition, due to their potentially transformational nature and associated cost, the evidence submitted to the HTA bodies is subjected to more intense scrutiny [18]. This could also be problematic with regard to the assessment of added clinical value for gene therapies in nAMD, considering that long-term outcomes addressing the HTA requirements may not be available at the time of launch. Moreover, the long-term benefits of a one-time administration of gene therapy vs. multiple anti-VEGF injections spread over time will not be fully realized in terms of the burden on patients, their caregivers, and healthcare systems.

At present, various HTA bodies, such as the National Institute for Health and Care Excellence (NICE), are employing traditional economic modeling or post-HTA evidence-generation mitigation strategies to handle the uncertainty regarding the long-term outcomes of gene therapies [17]. Although there have been advances in the way gene therapies are assessed by different HTA agencies, there is limited explicit guidance on how HTA agencies decide on their long-term benefits [17]. Explicit guidance on the assessment of gene therapies is also lacking at the EU HTA level. Specific considerations are rather limited in the JCA guidance documents and methodologies (related to the validity of clinical trials, endpoints, and applicability of evidence) [19].

It is anticipated that in the absence of long-term outcomes and/or appropriate comparisons (i.e., using matching external control arms/real-world evidence (RWE)/use of open-label LTFU etc.), the JCA may determine that there is significant uncertainty or even insufficient data for evaluating gene therapies or therapies for chronic diseases [19]. Thus, the lack of a suitable framework for assessing the durability of gene therapies in the JCA could significantly affect patients’ access to innovative treatments and may lead to disparities in access, particularly for gene therapies [20].

Therefore, the EU HTA framework should adopt customized solutions to establish the JCA as a valuable decision-making tool that improves patients’ access to innovative therapies [19]. In particular, a pragmatic approach should be adopted for the assessment of gene therapies, as methods developed for traditional pharmaceuticals may not be fit for purpose to address particularities related to the HTA of gene therapies [18]. HTA bodies should be open to adopting new methodologies to better manage the uncertainty regarding the long-term effectiveness estimates that gene therapies are anticipated to face. Moreover, the perspective of all relevant stakeholders and expert opinions should be considered to support durability arguments for gene therapies.

B. Discrepancies in outcomes between the RCTs and RWE available on comparators should be captured in JCA and reflected in the PICOs (population, intervention, comparators, and outcomes).

▪ Anti-VEGFs, which are the standard of care for the treatment of nAMD, demonstrate suboptimal results in real-world settings vs. the evidence from RCTs. This is mainly driven by the lack of adherence or differences in clinical settings across countries, leading to a reduced number of injections and/or active monitoring in the real world [10].
▪ JCA should allow for such discrepancies to be captured within the assessment and reflected in the PICOs to ensure that the most suitable comparative evidence is considered to guarantee optimal patient access, especially when the innovative therapy could help overcome these issues. Thus, JCA should consider evidence from indirect comparisons built using RWE to reflect clinical practice.

As previously mentioned, aVEGFs are the standard of care for the treatment of nAMD, and their efficacy has been demonstrated among various RCTs that investigate different dosing regimens [3,4,5,6,9]. However, the visual acuity outcomes obtained in RCTs are generally not replicated in clinical practice, as treatment regimens are typically more intensive and consistent in RCTs than in real-world clinical practice, which may also be variable between different clinical settings [10,21,22]. Annual injection frequencies reported in clinical practice demonstrate that most patients receive fewer injections than those participating in the clinical trials, often leaving their disease inadequately controlled. Consequently, the short-term and long-term visual outcomes of nAMD treatment in the real world are generally inferior to those seen in clinical trials of anti-VEGF therapies [10,21,22].

The disparity between outcomes achieved with nAMD management in clinical trials and real-world settings demonstrates an opportunity to improve treatment effectiveness. Additionally, the management of nAMD, with its need for frequent injections to prevent deterioration of visual acuity, places a substantial burden on patients, caregivers, and often over-stretched healthcare systems [22], especially considering the predominantly elderly population that suffers from the condition. These discrepancies between RCT outcomes and the real-world efficacy of aVEGFs should be reflected in the HTA assessment of gene therapies in nAMD.

RWE is broadly defined ‘as clinical evidence derived from analysis of real-world data related to patient health outcomes or experience, and/or treatment utilization/delivery in routine practice’ (incl. electronic health records or disease registries, etc.) [23]. For regulators, the main role of RWE is to inform long-term safety and effectiveness, and there have been several regulatory initiatives that enable the use of RWE during marketing authorization processes [24,25,26]. For HTA agencies, in addition to clinical outcomes, RWE helps to better understand the patient population, treatment pathways, comparators, and economic outcomes. RWE plays a crucial role in measuring treatment outcomes outside RCT settings.

In the context of EU HTAR, the assessment scope reflects policy questions from different healthcare systems using the population, intervention, comparator, and outcomes (PICO) framework [27]. The identified PICOs may vary across markets, and evidence from RCTs may not be sufficient to address all the different PICOs. RWE could be key to supplementing the evidence package and providing additional comparative evidence. As gene therapies undergo JCA since January 2025, it is essential to ensure that PICOs are informed by RWE, especially in case of gene therapies in nAMD, where long-term comparative RCT evidence may be limited at launch, and the outcomes of the current standard of care between RCTs and RWE settings are disconnected.

When generated in a robust way, RWE can provide a valuable source of evidence to support regulatory and HTA decision-making. The implementation of ‘good science’ principles is key to the successful conduct of RWE, including appropriate research design and valid traceable data [28]. The value of RWE is recognized by several HTA bodies, and advances have been made to broaden the use of RWE in the HTA processes. Published guidance from NICE [29] and Haute Autorité de Santé (HAS) [30] highlights that RWE has the potential to bridge evidence gaps by providing additional insights into the disease, its treatments, as well as comparative evidence to demonstrate the long-term value of the drug under assessment [23]. However, RWE is not systematically considered in HTA processes across the globe. The utilization of RWE in HTAs varies significantly by geography, and is often hindered by methodological challenges. These challenges are related to the reliability, acceptability, and generalizability of evidence, among other factors [31].

Moreover, there is a need for clear methodologies for the assessment of RWE within JCA. The Methodological Guideline for Quantitative Evidence Synthesis: Direct & Indirect Comparisons (hereunder referred as the ‘Guideline’) [32] highlights the statistical techniques that could be used for RWE to inform an estimate of relative effectiveness. However, the Guideline seems critical of such nonrandomized evidence, stating that *‘the certainty of the results provided by these techniques are controversial […]’ and ‘it may well be that this evidence is insufficient’* [32]. This approach could be problematic for the assessment of gene therapies in nAMD, considering the discrepancies observed between RWE and RCT outcomes of the current standard of care. Therefore, it is critical to ensure that indirect treatment comparisons using RWE will be considered during JCA and local HTA assessments.

C. Patient preference should be considered within the JCA, especially when assessing treatments involving single vs. repeated administration.

▪ The significant burden associated with anti-VEGF injections and the remaining unmet need highlights the need for more durable and effective therapies that can consistently maintain optimal outcomes in everyday clinical practice.
▪ Gene therapies in nAMD could advance patient management through optimizing clinical outcomes while simplifying service delivery.
▪ JCA should ensure that patient perspective regarding the frequency of administration is also considered during the assessment.

## 3. Conclusions and Additional Reflections

Frequent intraocular injections of anti-VEGF therapies have become the standard of care for nAMD. Effectiveness in the real world, however, often falls short of the outcomes demonstrated in clinical trials, resulting in loss of the visual acuity benefit initially observed during intensive anti-VEGF treatment. This suboptimal maintenance of long-term vision highlights the limitations and challenges of the existing real-world treatment paradigm. Gene therapies may represent a promising advancement in the treatment options for nAMD; however, several challenges may occur while assessing these new technologies through existing HTA methodologies. These challenges were discussed during the EAA breakout session in October 2024. The authors of this commentary are aligned with the key takeaways from the EAA breakout session and support the collaboration of multiple stakeholders for the development of an EU HTA framework suitable for the clinical assessment of gene therapies in nAMD. The authors also support leveraging LTFU, RWE, and indirect treatment comparisons in lieu of long-term and comparative evidence, and accounting for potential discrepancies between RCTs and clinical practice. Moreover, a clear fit-for-purpose framework will help to address policy questions that inform the PICO exercise.

## Data Availability

No new data were created or analyzed in this study.
